# Cost-Effectiveness of Telemedicine-Based Screening Programs for Retinopathy of Prematurity in Low- and Middle-Income Countries

**DOI:** 10.1016/j.xops.2026.101395

**Published:** 2026-09-04

**Authors:** Sarthak V. Shah, Arthur Brant, Michael R. James, Overbeck Christian Takou Mbah, Naana A. Wireko Brobby, Akwasi Ahmed, Sadik Teju Sherief, Cindy Zhao, Daniel Vail, Geoffrey Tabin, Joshua Salomon, Jeremy D. Goldhaber-Fiebert, Darius M. Moshfeghi

**Affiliations:** 1 Department of Ophthalmology, Duke University Medical Center, Durham, North Carolina; 2 Department of Ophthalmology, Byers Eye Institute, Horngren Family Vitreoretinal Center, Stanford University School of Medicine, Palo Alto, California; 3 Massachusetts Eye and Ear, Department of Ophthalmology, Harvard Medical School, Boston, MMassachusetts; 4 Department of Child Health, School of Medical Sciences, Kwame Nkrumah University of Science and Technology, Kumasi, Ghana; 5 Department of Ophthalmology, Komfo Anokye Teaching Hospital, Kumasi, Ghana; 6 Department of Ophthalmology, Addis Ababa University, Addis Ababa, Ethiopia; 7 Shiley Eye Institute, University of California San Diego, San Diego, California; 8 Department of Health Policy, Stanford University School of Medicine, Stanford, California

**Keywords:** Retinopathy of prematurity, Telemedicine, Cost-effectiveness analysis

## Abstract

**Objective:**

Evaluate the cost-effectiveness of telemedicine-based retinopathy of prematurity (ROP) screening strategies compared with binocular indirect ophthalmoscopy (BIO) and the current standard of care (no screening) in Sub-Saharan Africa.

**Design:**

Decision-analytic cost-effectiveness analysis simulating costs and vision outcomes across alternative ROP screening strategies.

**Subjects:**

A simulated cohort of 10 000 qualifying preterm infants (birth weight ≤2000 g or gestational age ≤32 weeks) in neonatal intensive care units across Ghana were modeled for each screening strategy.

**Methods:**

Three strategies were compared: no screening, BIO, and telemedicine-based screening with nonphysician graders. A hybrid telemedicine approach with 10% of images graded by physicians was also evaluated. The model estimated disease detection, treatment, and vision outcomes, and calculated costs and quality-adjusted life years (QALYs). Sensitivity analyses varied nonphysician grader specificity, ROP incidence, and physician evaluation time for BIO. A willingness-to-pay threshold of $2238 per QALY (1× Ghana's GDP per capita) was applied.

**Main Outcome Measures:**

Incremental cost-effectiveness ratios expressed as cost per QALY gained and dominance relationships among strategies.

**Results:**

Compared with no screening, telemedicine-based screening with nonphysician graders yielded health benefits at a cost of $27 per QALY gained. Incorporating 10% physician grading into the telemedicine platform further improved outcomes at $1331 per QALY gained relative to nonphysician grading alone. Binocular indirect ophthalmoscopy was strongly dominated by the hybrid telemedicine strategy. Sensitivity analyses identified nonphysician grader specificity, disease incidence, and physician evaluation time for BIO as the most influential parameters; overall conclusions were robust across plausible ranges.

**Conclusions:**

Telemedicine-based ROP screening, particularly a hybrid approach incorporating limited physician grading, appears to be a cost-effective strategy for implementation in Ghana and may be applicable to other countries in Sub-Saharan Africa.

**Financial Disclosure(s):**

Proprietary or commercial disclosure may be found in the Footnotes and Disclosures at the end of this article.

Every year, 32 300 babies worldwide develop irreversible blindness or visual impairment due to retinopathy of prematurity (ROP).^1^ Blindness from ROP is preventable with treatment; infants go blind because they were not examined in a timely manner. Blindness can, in some cases, severely damage quality of life, often exacerbated by the contexts of low- and middle-income countries (LMICs), where the consequences can include up to 50% mortality rate during early childhood, higher rates of parental abandonment, inability to work, and a lifelong requirement for caregivers.^2^

Neonatal care in Sub-Saharan Africa (SSA) is rapidly expanding with increased access to oxygen and basic medical equipment. In Ghana, 128 000 babies are born preterm annually.^3^, ^4^, ^5^ Fortunately, treatment with intravitreal bevacizumab, is widely accessible throughout the country, costing less than $30 per eye.^6^ Despite treatment availability, screening remains the limiting factor and constitutes most of the work for an ROP program.

Screening is commonly limited by scarcity of ophthalmologists, lack of expertise, and competing clinical priorities. In Ghana, there are only 12 retina specialists or pediatric ophthalmologists serving 34 million people versus >3000 in the United States serving 340 million. Often faced with a difficult disease to diagnose (i.e., ROP) versus highly prevalent diseases that require chronic management (e.g., diabetes and sickle retinopathy), screeners gravitate toward the latter. This is unfortunate in that ROP represents a contained disease where timely identification and solitary intervention within 24-48 hours of diagnosis confers lifelong benefit. Most countries in SSA lack screening guidelines.^7^^,^^8^

There are 2 main techniques for ROP screening (Table S1, available at www.ophthalmologyscience.org): Binocular indirect ophthalmoscopy (BIO), the putative gold standard,^9^^,^^10^ and telemedicine using wide-angle digital retinal imaging (TeleROP).^11^ It needs to be acknowledged *a priori* that in ROP screening, experience does not equate with expertise, and sufficient understanding of disease features is necessary to accurately detect an appropriate inflection point for intervention.

Binocular indirect ophthalmoscopy is predicated upon an in-person examination using special equipment to view the retina—an indirect ophthalmoscope, a condensing lens, a speculum, and a scleral depressor—to obtain 360° visualization of the retina after pharmacologic dilation. This requires mentored specialty training after medical school, ophthalmology residency. Telemedicine using wide-angle digital retinal imaging leverages ROP expertise to the task at hand—assessing for treatment-warranted ROP (TW-ROP) defined as type 1 disease per the early treatment for ROP trial or aggressive ROP^12^^,^^13^—as opposed to the totality of the ROP screening and grading enterprise.^11^ In comparison to BIO which necessitates substantial physician time for commute and examination, TeleROP offloads screening to nonphysician expert fundus photographers. Images are then remotely graded by a physician or some combination with an expert technician grader. This limits time-consuming in-person BIO examinations to the small subset of patients with incidental lesions, ungradable photos, or treatment-warranted disease.

Telemedicine using wide-angle digital retinal imaging screening programs are a promising solution to common constraints in LMICs. TeleROP has been validated in the United States, India, and LMIC settings proving to be safe and efficacious.^11^^,^^14^^,^^15^ In summer of 2024, Komfo Anokye Teaching Hospital (KATH) launched a fully telemedicine-based screening program for ROP in Kumasi, Ghana, serving a 10-M catchment population in the Ashanti Region. Since its initiation, this program screens >1000 babies per year and accepts referrals from >20 level II and III neonatal intensive care units covering a total of 400 neonatal beds. Retinopathy of prematurity screening is performed by a trained photographer with the Forus 3nethra NEO 120-degree camera.^16^ If TW-ROP is identified, treatment by an ophthalmologist occurs within 24-48 hours as urgent care is critical to minimize the chances of any development of vision impairment. The screening criteria used in Kumasi, Ghana is ≤2000g birth weight or gestational age of ≤32 weeks, with the first eye examination at 2 weeks of life or discharge, whichever is earlier. These criteria are based on existing West African ROP screening programs and are intentionally broad to be inclusive of larger babies at risk until further validation is performed in the Ghanaian population.^6^^,^^17^

Despite the promise of TeleROP in LMICs like Ghana, there is sparse literature on the cost-effectiveness of this intervention. Hence, we perform a model-based economic evaluation of screening for ROP in a simulated patient population in Ghana.

## Methods

### Decision-Analytic Model

Decision-analytic and Markov models were created and analyzed in Python 3.13.1 (PyCharm environment). The parameters for probabilities, costs, and utilities are detailed in Table 1.

**Table 1:** Key Input Parameter Values, Ranges, and Sources

Parameter	Value	Range	Source
Population			
Ghana annual preterm births, per year	128 000		^5^
Incidence of TW-ROP	0.05	0.03–0.30	^6^^,^^16^^,^^18^KATH
Ghana life expectancy, years	65	63–69	^19^^,^^20^
TW-ROP recurrence after treatment	0.02	0.02–0.10	^21^^,^^22^, KATH
Ghana annual probability of death by age	Age-dependent		^19^
Treated TW-ROP end-state vision probability			
No impairment	0.674		^13^^,^^22^
Mild impairment	0.213		^13^^,^^22^
Moderate impairment	0.079		^13^^,^^22^
Severe impairment	0.034		^13^^,^^22^
Blindness	0	0–0.13	^13^^,^^22^
Untreated TW-ROP end-state vision probability			
No impairment	0.28		^23^
Mild impairment	0.03		^23^
Moderate impairment	0.1		^23^
Severe impairment	0.12		^23^
Blindness	0.47	0.41–0.54	^23^
Treatment-related complication rates			
Cataract	0.0015	0.0015–0.095	24, 25, 26
Rhegmatogenous retinal detachment	0.001		^26^
Endophthalmitis	0.0007		^26^
Grading efficacy			
Image readability	0.99		KATH
Ophthalmoscopy			
Sensitivity	0.99	0.75–0.999	^27^
Specificity	0.99	0.75–0.999	^27^
Telemedicine graded by physician			
Sensitivity	1	0.75–1.00	^11^^,^^16^^,^^27^, ^28^, ^29^
Specificity	0.98	0.75–0.99	^11^^,^^16^^,^^27^, ^28^, ^29^
Telemedicine graded by nonphysician			
Sensitivity	0.99	0.75–0.99	^16^, KATH
Specificity	0.95	0.75–0.99	^16^, KATH
Costs			
Ophthalmoscopy evaluation, $USD per case	9.38	6.25–70	^30^, KATH
Ophthalmoscopy equipment, $USD per set	3259		^30^
Telemedicine setup, $USD per 1000 cases/year for 10 years	35 000		KATH
Time			
Ophthalmoscopy screening and grading time, min per case	45	30–336	Expert consultation
Telemedicine screening, min per case	5	3–10	KATH
Telemedicine grading, min per case	1	5–10	KATH
No. of follow-up examinations for treated babies	3	2–4	KATH
No. of telemedicine examinations	6	2–8	KATH
Ghana average workforce labor time, hours per year	2400	2200–4160	KATH
Salary, $USD per person per year			
Physician	30 000	30 000–80 000	KATH
Telemedicine screening technician	5000	2700–6000	KATH
Treatment cost, $USD per case			
Anti-VEGF	60		KATH
Complication: cataract	300		KATH
Complication: retinal detachment	1000		KATH
Complication: endophthalmitis	1000		KATH
Vision impairment utility			
No impairment (20/40)	1.000	0.89–1.00	^31^^,^^32^
Mild impairment (20/60)	0.997	0.700–0.997	^31^^,^^32^
Moderate impairment (20/200)	0.969	0.64–0.98	^31^^,^^32^
Severe impairment (20/600)	0.50	0.50–0.88	^31^^,^^32^
Blindness	0.50	0.50–0.88	^31^^,^^32^

KATH = information collected via surveys, interviews, and retrospective analysis from Komfo Anokye Teaching Hospital; TW-ROP = treatment warranted retinopathy of prematurity.

Costs provided in 2025 USD.

### Target Population

The target population for this analysis includes preterm infants in Kumasi, Ghana, admitted to the KATH neonatal intensive care units and meeting the ROP screening criteria of birth weight ≤2000 g or gestational age ≤32 weeks. In line with current volumes (1000 infants/year), we simulated outcomes for 10 screening centers (10 000 infants). The incidence of TW-ROP among screening-eligible infants is estimated to be approximately 5% based on a consensus and the clinical experience of local physicians (A.A. and N.A.W.B.). Institutional review board approval was given by KATH, and the study was deemed exempt by the Stanford institutional review board for collection of baseline cost and incidence parameters: AMP1/201/24. Informed consent was obtained from parents of all enrolled infants. The Declaration of Helsinki and human research standards were followed, and all methods and protocols were carried out in accordance with regulations as set by the institutional review board.

### Study Perspective, Discount Rate, and Time Horizon

This analysis adopts the health care sector perspective, focusing on direct medical costs associated with implementing and maintaining a telemedicine-based ROP screening program excluding indirect costs such as caregiver time or productivity losses. An 85-year lifetime horizon with a 1-year cycle length to capture annual transitions between health states (e.g., treated, untreated, or blind) was selected to fully track the lifelong economic and quality-of-life consequences of childhood blindness. This horizon extends 20 years beyond the current national average life expectancy in Ghana to properly capture the full distribution of the cohort's survival, as individuals exit the Markov model probabilistically based on age-specific mortality rates rather than truncating uniformly at the mean life expectancy of 65 years. An annual discount rate of 3% is applied to both costs and outcomes to reflect the present value of future benefits. To evaluate the impact of long-term macroeconomic uncertainty and promote intergenerational equity in alignment with contemporary cost-effectiveness guidelines, alternative annual discount rates of 1.5% and 0% were evaluated in sensitivity analyses.

### Screening Strategies

The model evaluates 3 primary screening strategies (Fig 1). (1) The current Standard of Care (SOC) strategy in Ghana: no screening and no treatment. (2) The BIO strategy: screening and grading are performed by a physician. (3) The base unassisted telemedicine (TeleROP) strategy: screening and grading of all images are performed by a trained nonphysician technician. Follow-up analysis evaluates a hybrid telemedicine strategy with a portion of physician grading (TeleROP n% Phys) where screening of all images is performed by a trained nonphysician technician,^24^ but grading responsibility is split. A random subset of 10%-100% is modeled to be graded by a physician, with the remainder by a nonphysician technician.

1. Standard of care

**Figure 1:**
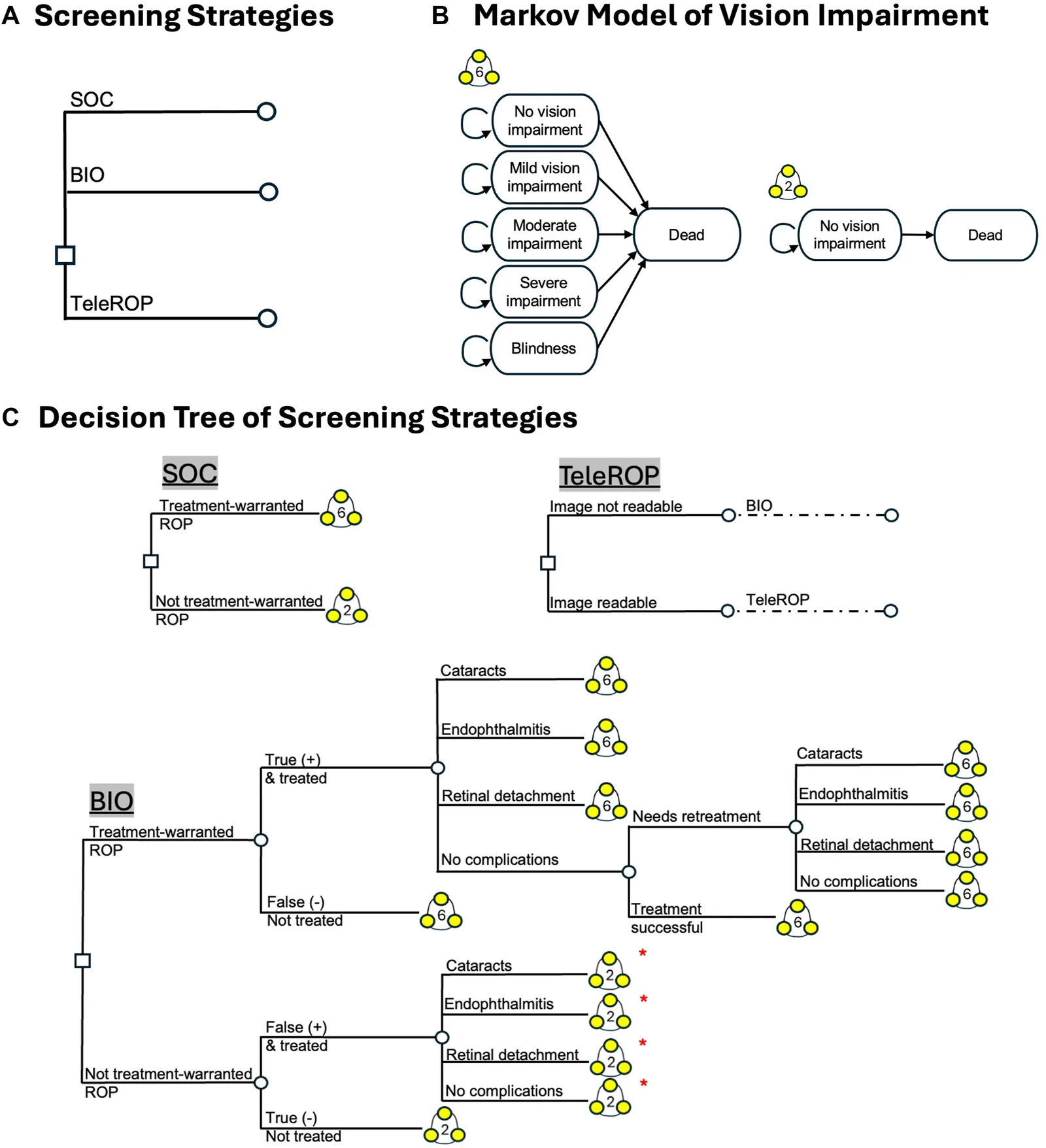
Model schematics. **(A)** For the screening strategies, SOC represents Standard of Care which equates to no screening. BIO represents the screening strategy with binocular indirect ophthalmoscopy. TeleROP represents the screening strategy of telemedicine, whereby both screening and grading of all images are performed by a nonphysician technician. TeleROP n% Phys represents the screening strategy of telemedicine, whereby screening of all images is done by a nonphysician technician but grading of n% of screened images is performed by a physician, and grading of 100-n% of screened images is performed by a nonphysician technician. **(B)** Depiction of decision tree for all 4 screening strategies chosen to be compared. **(C)** Markov model used to calculate final costs and benefits over the average Ghanian lifespan. The mini state-transition diagrams with yellow nodes indicate the number of states in the Markov model. ∗ Individuals without treatment-warranted ROP who test positive and are treated (false (+) and treated) may develop complications from the treatment. However, complications generally affect only 1 eye, and vision utilities are based on that of the better-seeing eye. Therefore, regardless of complications, these individuals have no permanent visual impairment. BIO = binocular indirect ophthalmoscopy; ROP = retinopathy of prematurity; SOC = Standard of Care.

Individuals in the SOC strategy either have TW-ROP or do not. Those with no TW-ROP have no vision impairment and are followed over their lifetimes facing annual age-specific risks of death. Those with TW-ROP in this strategy do not receive screening or treatment. Therefore, they have varying degrees of vision impairment and are followed over their lifetimes; each year they remain with their level of vision impairment and face annual age-specific risks of death. Given the permanent nature of the disease determined in the first 6 months of a neonate's life, transition between states of vision impairment was not allowed (Fig 1).

2. BIO strategy

Those with no TW-ROP in the BIO strategy can either test negative (true negative) or positive (false positive). Those who test negative are not treated remain with their level of vision impairment each year and face annual age-specific risks of death. Those who test positive are assumed to all receive subsequent treatment in our model. Treatment confers a very low risk of complications including cataracts, endophthalmitis, or retinal detachment. Complications with long term vision complications generally affect only 1 eye, and vision utilities are based on that of the better-seeing eye.^25^^,^^26^ Therefore, individuals with complications that cause a one-time increase in costs remain with their level of vision impairment each year and face annual age-specific risks of death along with those without complications. Those with TW-ROP in this strategy can either test negative (false negative) or positive (true positive). Those who test negative do not receive treatment have varying degrees of vision impairment and are followed over their lifetime as described in the SOC section. Those who test positive receive treatment with an associated low risk of complications. These individuals have varying degrees of vision impairment and are followed over their lifetimes. We assumed that those without complications after treatment can either have a successful treatment or a risk of reactivation.^21^^,^^33^ This reactivation would require retreatment, generally done once, with associated complications (Fig 1).

3. Base unassisted telemedicine strategy

The majority of images taken by the camera are readable and will continue through the TELE decision tree and Markov model. This model is similar to the BIO's described earlier, with the main difference being the diagnostic accuracy of the screener, grader, and associated costs. A minority of images will not be readable and screening will be done through BIO (Fig 1).

### Probabilities

Estimated probabilities and ranges come from peer-reviewed clinical publications and observational data from screening centers in Accra and Kumasi, Ghana.

#### Incidence of Disease

Incidence of TW-ROP among screening-eligible infants (≤32 weeks gestation or ≤2000g birth weight) was assumed to be 5% based on collected data from KATH. Because incidence of ROP varies widely in countries with unfractionated oxygen burden, sensitivity analysis ranges from 1% to 30%.^6^^,^^16^^,^^18^

#### Image Quality

For telemedicine-based screening strategies, fundus image readability is critical; although triaging for the inflection point of treatment warranted ROP is often possible with limited or sub-par image quality. To acknowledge the potential limitation where all photos are ungradable per eye visit (ex. corneal opacity/cataract/vitreous hemorrhage/congenital glaucoma), we modeled a portion of telemedicine examinations that would need BIO examination. We estimated that visits were gradable with strictly telemedicine 99% of the time from clinical experience at KATH and that uninterpretable examinations required ophthalmoscopic screening. Sensitivity analyses included alternate probabilities as low as 95% interpretability.^14^^,^^34^

#### Sensitivity/Specificity of Screening Strategies

The sensitivity and specificity of different screening strategies are estimated based on validated clinical trial programs, incorporating hybrid approaches where nonphysician technician graders evaluate the majority of images, with ophthalmologists reviewing the remainder. To approximate the sensitivity and specificity of hypothetical 100% technician graders in the base case of telemedicine based on tested sensitivity and specificity of technician graders in the GHANAROP program. Sensitivity analyses also included values from technician graders from the published KIDROP model.^16^ The sensitivity and specificity of 100% graded telemedicine-based screening come from published values of SUNDROP.^11^ Approximate hybrid models of ROP screening with nonphysician technician photographer graders grading a given proportion of the images were calculated via weighted averages of sensitivity and specificity values. Notably, telemedicine-based screening demonstrates superior sensitivity than traditional ophthalmoscopy, primarily due to the image comparability available to telemedicine graders. In contrast, real-time ophthalmoscopy relies on physicians' memory or handwritten notes to conduct the grading process, which may limit consistency and accuracy.

### Costs

Costs were expressed in 2025 US dollars.

#### BIO

Ophthalmoscopic screening costs include professional and facility fees. Examination time was estimated at 45 minutes, including transport, documentation, and preparation time. Cost per minute worked was estimated with mean ophthalmologist salary in Ghana.

#### Telemedicine

Telemedicine expenses include a $35 000 equipment setup cost (ten $30 000 cameras with $5000 additional cost for maintenance and repair per camera, divided over 10 years due to the average lifespan of the device to get the camera costs per year) in order to screen 10 000 babies annually (1000 babies per year/each center). Based on a modeled throughput of 1000 infants screened per camera annually (equating to approximately 6000 individual examinations per year, or roughly 115 examinations per week), the capital assets would require near full-time, dedicated clinical utility, thereby precluding cost-sharing or cost-allocation with other ophthalmic services. The model also accounts for ongoing costs from technician salaries and cost for image grading based on the proportion of physician grading, with each examination requiring about 5 minutes (Table 1). Redundancy and expertise are very important for screening and grading. Thus, salaries for technicians and physicians are split between 2 persons for each role to allow for coverage 365 days/year. Costs for training for technicians is a one-time cost, and was left out of the modeled analysis. Structure technician training followed the structure of previously published eROP and KIDROP programs.^16^^,^^28^

#### SOC

No additional costs were modeled in this group.

#### Number of Examinations

Ophthalmoscopic screening interval is generally determined by the severity of disease, with a mean of 4 examinations/patient. In GHANAROP, patients have a mean of 6 telemedicine examinations. A subset of patients that require treatment receive an additional mean of 3 follow-up examinations for each treatment.

#### Treatment

Treatment with intravitreal bevacizumab injections for both eyes was modeled to be $50, and are inclusive of the medication, consumables, and physician time. Treatment costs are based on clinical practice patterns at major Ghanaian tertiary medical centers—including KATH, Korle-Bu Teaching Hospital, Greater Accra Regional Hospital, and Tamale Teaching Hospital. Intravitreal bevacizumab was modeled as the sole primary intervention (100% of treated cases). Laser therapy was explicitly excluded from the primary model structure due to its limited local utilization, high equipment procurement/maintenance barriers, and the clear clinical predominance of anti-VEGF monotherapy in this setting.

#### Complications

One-time costs from retinal detachment, cataracts, and endophthalmitis resulting from treatment were modeled.

#### Unmodeled Costs

Liability costs are an important indirect cost but are not yet applicable in Ghana and were not included.

### Health Outcomes and Quality of Life

Health outcomes were measured in quality-adjusted life years (QALYs). Utility weights were assigned for infants in 5 states with no visual impairment, mild impairment, moderate impairment, severe impairment, and blindness. Proportions of infants ending in each utility state were modeled based on treatment status. Untreated infants with TW-ROP were modeled to have vision outcomes as published in the natural history arm of the CRYOROP study.^23^ The utilization of CRYOROP data to estimate untreated visual outcomes represents a structural limitation due to unavailability of natural history data in LMIC contexts. Because of profound differences in neonatal care infrastructure and infant population baseline risks between historical US cohorts and contemporary Ghanaian settings, our model may systematically underestimate the true clinical severity and downstream lifetime disutility of untreated ROP within the Sub-Saharan African context. Correctly treated infants were modeled to have vision outcomes as published in the bevacizumab arm of the ETROP trial.^13^ Published studies have quantified health utilities for different levels of visual acuity using methods such as the time trade-off and standard gamble approaches. There is a wide discrepancy in the literature for the disability weights for vision impairment, and thus both the best and worst case were modeled. For this analysis, we extracted best-case utility values for visual impairment and blindness from the Global Burden of Disease Study, published in The Lancet Global Health in 2019.^31^ The utility values from the Global Burden of Disease study represent the global health–related quality of life impact of varying degrees of vision loss and are widely used in ophthalmology cost-effectiveness literature. For the worst-case utility values we converted Snellen visual acuity values based on a published formula: utility = 0.374x + 0.514, where x represents Snellen visual acuity in decimal format.^35^

### Sensitivity Analyses

Sensitivity analyses assessed the robustness of the findings to changes in incidence of TW-ROP, probability of blindness with and without treatment, complication rates, image readability, sensitivity and specificity for physicians and technicians, grading and screening time, number of evaluations, quality of life adjustments for vision impairment, treatment costs, and reactivation rate. Follow-up assessments of hypothetical physician/technician hybrid telemedicine programs from 10% to 100% physician graded examinations were assessed. One-way sensitivity analyses were performed to classify thresholds of technician grader sensitivity and specificity for which BIO had dominance over telemedicine strategies. Two-way sensitivity analyses with technician specificity and incidence were used to understand the effects of possible overtreatment on preferred screening modality. Threshold analyses were based on a conservative willingness-to-pay threshold of $2238 per QALY gained, reflecting 1x of Ghana's 2025 GDP per capita.

## Results

For a modeled cohort of 10 000 preterm infants, the total costs and QALYs accrued by each strategy are summarized in Table 2. In the worst case for vision impairment disability weights,^35^ the incremental cost-effectiveness ratio (ICER) for telemedicine screening with nonphysician graders compared to no screening was $27 per QALY gained. Incorporating 10% physician grading into the telemedicine strategy resulted in an ICER of $1331 per QALY gained compared to telemedicine with nonphysician graders alone. The BIO strategy was strongly dominated by telemedicine with 10% physician grading, offering fewer QALYs at greater cost. Even if vision loss results in less disutility, our findings remained of which strategies remained on the efficient frontier were robust (Table 2; Fig 2).

**Table 2:** Costs, Quality-Associated Life Years, and Incremental Cost Effectiveness Ratios by Strategy Grouped by Best- and Worst-Case Utility Values for Blindness

Strategy	Costs ($)	QALYs		ICERs ($/QALY)	
Utility case	Both^24^^,^^25^	Best^24^	Worst^25^	Best^24^	Worst^25^
Standard of care	0	272 341	269 807	-	-
Telemedicine screening (nonphysician)	102 835	273 748	273 577	73	27
Telemedicine screening (10% physician)	107 854	273 749	273 581	3566	1331
Ophthalmoscopy	426 414	273 748	273 577	Dominated	Dominated

ICER = incremental cost effectiveness ratio; QALYs = quality associated life years.

Dollar amounts in 2025 USD for 10 000 patients.

**Figure 2:**
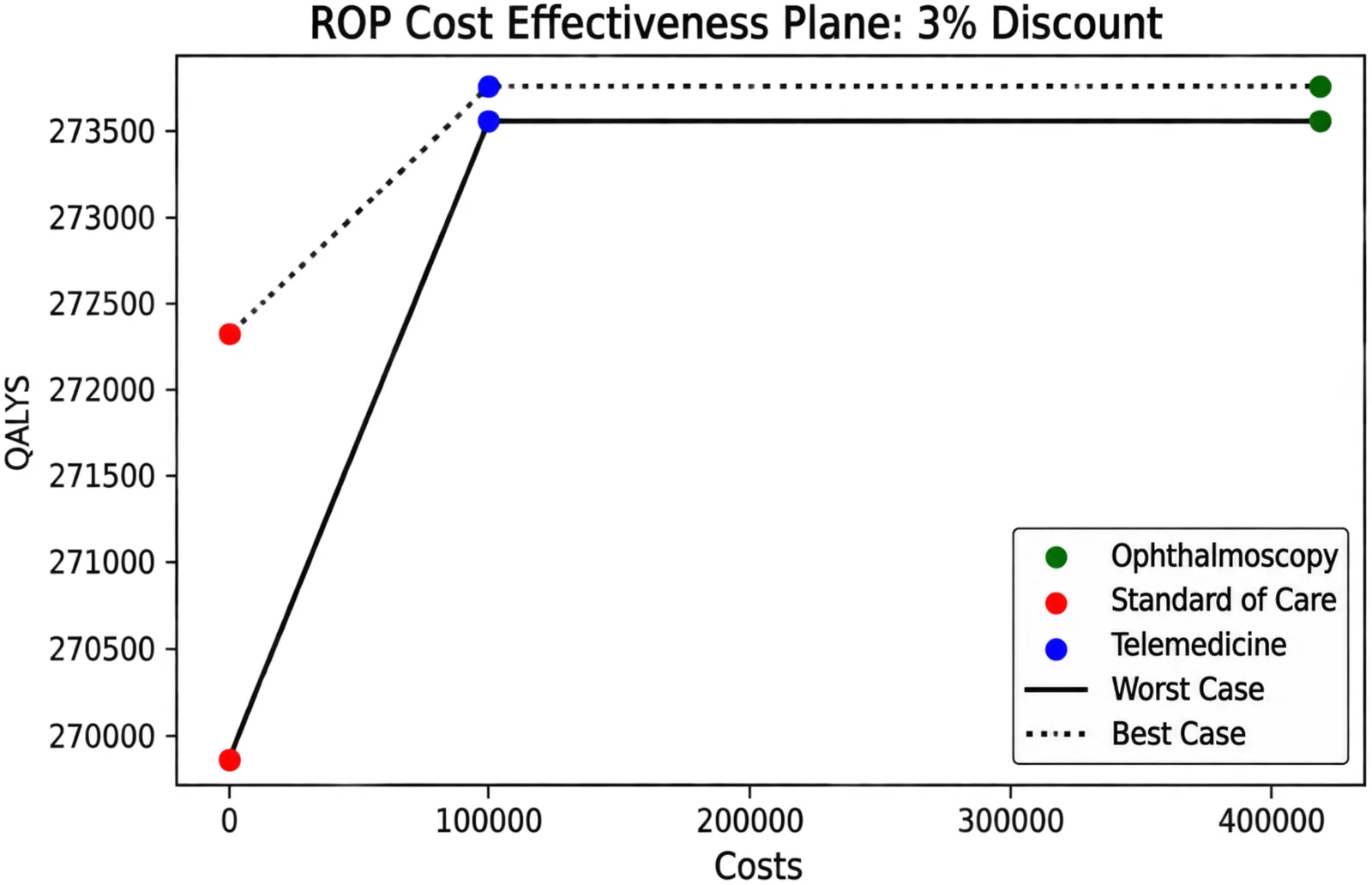
Cost-effectiveness plane, including standard of care, TeleROP with 100% nonphysician grading, and ophthalmoscopy for worst- and best-case utility values for vision impairment. Costs are expressed in 2025 USD. QALYs = quality adjusted life years; ROP = retinopathy of prematurity.

When assessing the percentage inclusion of physician grading into the TeleROP platform, costs and QALYs rise in an apparent linear fashion (Fig S1, available at www.ophthalmologyscience.org) inspired by hybrid grading approaches used in LMICs.^16^

Sensitivity analysis confirmed the robustness of these findings, with cost and QALY estimates varying primarily with the utility assigned to vision impairment. Across simulations, telemedicine-based strategies consistently incurred lower costs than BIO, even when physician evaluation costs were minimized. When conducting 1-way sensitivity analyses (Fig S2, available at www.ophthalmologyscience.org), TeleROP with 10% physician graders remains the most cost-effective strategy across the entire range of most parameters. Which strategy was most cost-effective was most sensitive to the sensitivity of telemedicine nonphysician graders, the sensitivity and specificity of physician graders and utility values for blindness. Lower discount rates of 1.5% and 0% strengthened existing trends and did not change resulting dominance of telemedicine screening strategies (Table S2, available at www.ophthalmologyscience.org).

Follow-up single parameter sensitivity analyses showed that when the sensitivity of technician graders dropped below 94%, BIO became the preferred strategy. The preferred strategy did not change for any variation in technician specificity (Fig S3, available at www.ophthalmologyscience.org).

Two-way sensitivity analysis indicated that only when disease incidence was low (<4%) and when technician specificity was high (>96%), was TeleROP with nonphysician graders preferred to TeleROP with 10% physician grading. Further 2-way sensitivity analysis on technician grading accuracy showed the findings preferring TeleROP with 10% physician graders was more sensitive to grader sensitivity versus grader specificity (Fig S4, available at www.ophthalmologyscience.org).

Additional 2-way sensitivity analyses show that the cost-effectiveness of the telemedicine strategy with 10% physician graders compared to SOC is not very sensitive to technician grader accuracy and ROP incidence. Within our value ranges, no combination of technician specificity and ROP incidence (Fig 3A) or technician specificity and sensitivity (Fig 3B) resulted in an ICER of more than $100/QALY.

**Figure 3:**
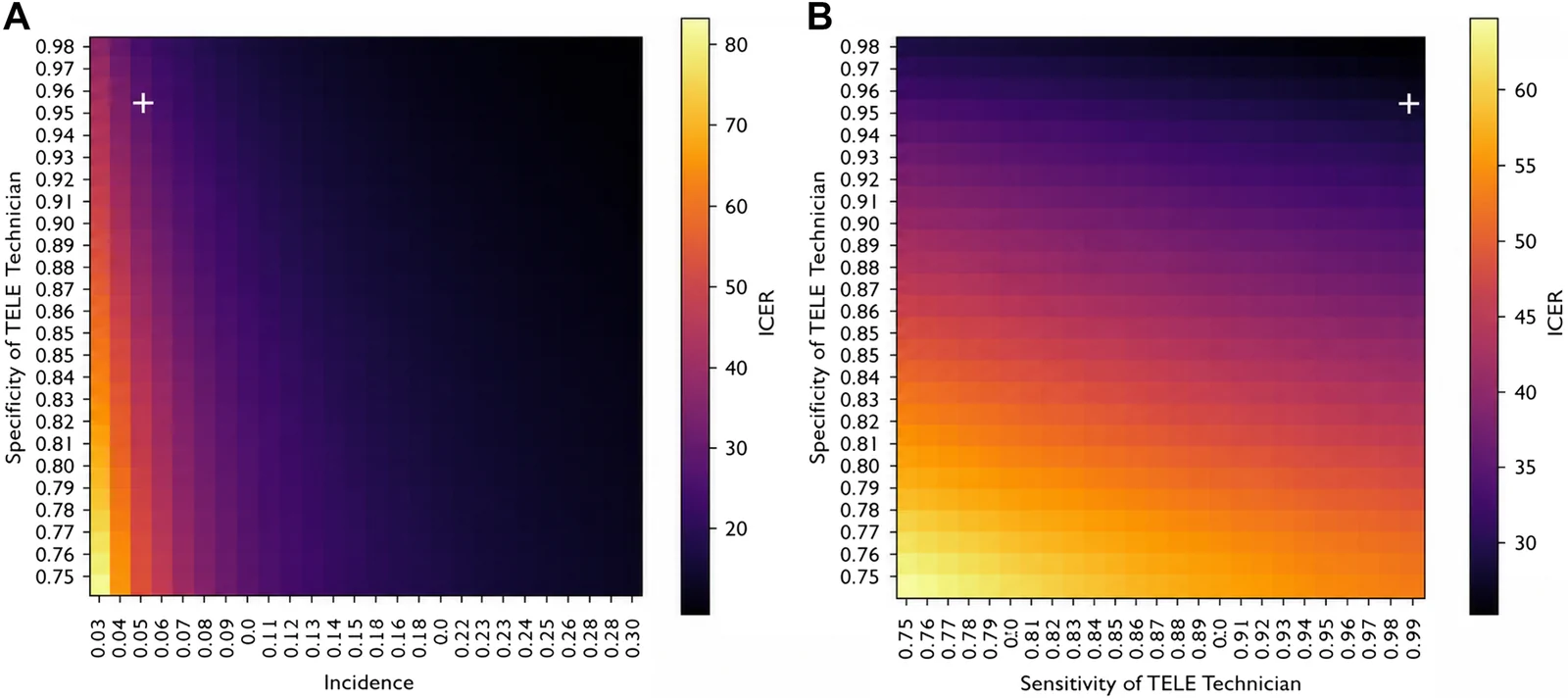
Heat maps plotting the incremental cost-effectiveness ratio (ICER) of the telemedicine strategy with 10% physician graders compared to standard of care varied along 2 parameters: **(A)** specificity and sensitivity of technician grader and **(B)** specificity of technician grader and ROP incidence. The white “plus” indicates the base case. ROP = retinopathy of prematurity.

## Discussion

Our cost-effectiveness analysis of ROP screening strategies in Ghana reveals several key findings. Telemedicine-based screening with nonphysician graders demonstrates dominance over the current SOC (no screening), with an ICER of $27 per QALY gained. Incorporating 10% physician grading into the telemedicine platform further improves outcomes, costing $1331 per QALY gained compared to nonphysician grading alone. Binocular indirect ophthalmoscopy was strongly dominated by telemedicine with 10% physician grading.

Sensitivity analyses indicate that the findings preferring TeleROP with 10% physician graders are generally robust but also highlight that the findings are sensitive to variations in the sensitivity of nonphysician graders, grading accuracy of physician and utility for blindness. When technician grader sensitivity falls below 94%, BIO is preferred. At lower disease incidence rates (below 4%) combined with improved technician sensitivity, entirely well-trained nonphysician telemedicine graders emerge as the preferred strategy. These thresholds underscore the importance of maintaining high-quality training programs for nonphysician graders and accurately determining the true incidence of TW-ROP in Ghana. A probabilistic sensitivity analysis was intentionally omitted from this study. Given the current paucity of robust, primary epidemiological and operational data from ROP programs across SSA, assigning arbitrary probability distributions (e.g., beta or gamma distributions) to highly uncertain parameters risk introduction of false precision. Instead, we relied on comprehensive 1-way and 2-way deterministic sensitivity analyses spanning wide, clinically plausible ranges. Because this deterministic approach successfully isolated clear, actionable policy decision thresholds, a probabilistic sensitivity analysis was deemed unnecessary and structurally counterproductive for local decision-making.

Improvements in artificial intelligence algorithms for ROP diagnosis and triage may further improve the cost effectiveness of a telemedicine-based model.^36^ Currently, there are no US Food and Drug Administration approved algorithms to diagnose TW-ROP or referral-warranted ROP and thus we have elected to not include this in the study. Once commercially available models become available and can be validated for clinical practice in LMICs, we think it would be promising to model cost-effectiveness in LMICs with resulting sensitivities and specificities.

Our results are broadly consistent with the findings of Morrison 2022^14^ that telemedicine-based ROP screening is a more cost-effective strategy than traditional ophthalmoscopy despite differing economic contexts. Notably our analysis used Ghana's GDP per capita ($2238) as the willingness-to-pay threshold instead of $100 000 per QALY gained for Morrison 2022. Our overall BIO costs were also notably less than a comparable study conducted on cost-effectiveness of BIO in Rwanda, likely representing differences in local costs.^30^ Despite this conservative assessment, our study showed the costs of telemedicine strategies were roughly one-fourth the cost of BIO strategies. Sensitivity analysis revealed that this cost dominance of telemedicine persists across all tested parameters. It is interesting to note the proportional cost delta of TeleROP versus BIO in the United States (about 50% savings) in comparison to LMICs (about 75% savings) with only physician graders.^37^ The United States has 42.5x the GDP per capita of Ghana, this is important to consider when comparing costs. The absolute cost/QALY for TeleROP in LMICs modeled is 100x less than the cost/QALY in the United States.^33^ With regard to scaling potential, BIO scaling is capped by availability of physician screeners. In comparison, TeleROP with predominantly technician graders theoretically grows superlinearly based on numbers of cameras and technicians (assuming availability of labor and funds for cameras).

Our analysis adopts the health care sector perspective, which does not account for the broader societal impacts, such as the economic burden on families caused by childhood blindness. This thereby excludes significant indirect societal costs such as caregiver time, travel, and productivity losses. Clinical experience in Ghana indicates that infant ROP screening visits are synchronized with SOC pediatric weight checks which effectively minimizing unique travel costs exclusive to screening. The model also does not formally account for transportation-related loss to follow-up. Future studies incorporating a societal perspective could better capture these indirect costs and potential additional benefits of averting blindness.

Key assumptions in this study, such as the sensitivity and specificity of technician graders in Ghana, are based on data from the validated KIDROP program in India.^16^ Although this serves as a useful proxy, the true accuracy of these graders in Ghana remains uncertain. Furthermore, our model simplifies the hybrid screening framework by simulating a randomized escalation pattern. In clinical environments, escalation is inherently grader-dependent, dictated by individual risk tolerance, clinical experience, and varying infant image quality. Actual clinical implementation may therefore introduce variable triage behaviors not fully captured by our conservative randomized simulation. Additionally, the current decision-analytic model does not account for inter-reader and intrareader variability among technician or physician graders. While our baseline parameters reflect robust diagnostic performance extracted from established overseas models, prospective multicenter studies within the West African health care workforce are required to map the exact distribution of true reader variability. Another uncertainty lies in the actual incidence of TW-ROP in Ghana, which is an ongoing study and the data have yet to be firmly established. Lastly, the deliberate exclusion of laser photocoagulation from our economic model reflects specific operational treatment realities in Ghana, but it may limit the immediate generalizability of these findings to other Sub-Saharan African networks where laser remains a primary therapeutic modality. Although this exclusion affects the total cost output of the model, it does not preferentially impact 1 screening modality over another and would not change the conclusions of the preferred screening strategy.

Despite these limitations, this study provides compelling evidence for the implementation of telemedicine-based ROP screening programs in Ghana and other LMICs. At $27 per QALY gained, telemedicine for ROP screening and treatment is 1 of the most cost-effective programs in ophthalmology, especially in physician limited settings where BIO screening is impractical. As neonatal care continues to advance in SSA, the integration of cost-effective ROP screening strategies will be integral to prevent unnecessary childhood blindness. The decision-analytic framework developed in this study is highly adaptable and readily transferable to other national health systems across SSA. By replacing the model's localized baseline inputs (ex. regional ROP incidence rates, country-specific physician and technician salary structures, and local equipment procurement and contract maintenance fees), policymakers can easily recontextualize this cost-utility frontier to guide regional blindness-prevention allocations.
