# Supplementary material for: Cost-Effectiveness of Telemedicine-Based Screening Programs for Retinopathy of Prematurity in Low- and Middle-Income Countries
Source: Ophthalmol Sci. 2026 Sep 4;6(11):101395. doi: 10.1016/j.xops.2026.101395 (PMC13654025; doi:10.1016/j.xops.2026.101395)
Supplement: Supplementary file 1 [file mmc1.pdf]

Supplemental Table 1. Overview of Screening Methods for Retinopathy of Prematurity

**Binocular Indirect Ophthalmoscopy (BIO)**	**Telemedicine (TeleROP)**
Workflow	
- Qualifying infant is dilated<br>- Speculum is inserted<br>- 360° binocular indirect ophthalmoscopy is performed with scleral depression in the first eye (1-5 minutes)<br>- Findings are documented on a schematic drawing<br>- Findings are interpreted to achieve one of three outcomes (e.g., repeat screening, treatment, termination of acute phase screening)<br>o In the event of repeat screening, follow-up interval is determined by the findings (e.g., Zone/Stage/Plus combinations dictated whether the infant will be seen in <1 week, 1 week, 2 weeks, 3 weeks or more)<br>- Report is filed or entered into the medical health record system<br>- Next week, some or all of the infants are examined<br>- Screeners may rotate weekly/monthly/quarterly	- Qualifying infant is dilated<br>- Speculum is inserted<br>- Trained photographers obtain a standard image on each eye of each infant at each exam.<br>- Images are synchronized with a cloud-based, HIPAA-compliant secure location.<br>- Images are then remotely graded by a physician or some combination with an expert technician grader, decreasing the physician time per patient.<br>o Disease severity can be assessed with the Telemedicine for Retinopathy of Prematurity Severity Score (tROP-SS). The tROP-SS integrates the 3 variables of ROP (Zone, Stage, and Plus) –and returns a single value that can be tracked relative to the intervention level (≥55 points). Any person, regardless of medical training, can assess the tROP-SS as it is plotted versus the intervention point and determine both infant's risk of treatment and relative status.<br>- A mandated screening interval is employed resulting in more screening visits per patient on average compared to BIO. Intervals are mandated to be weekly or more often, depending on disease severity until the infant reaches 45 weeks post menstrual age.
Strengths	
- Three-dimensional stereoscopic viewing<br>- Ability to see 100% of the retina with scleral depression	- Objective documentation<br>- Objective longitudinal comparison<br>- Ability to perform quality assessment/improvement based on the imaging

- Screening and grading are done by the same examiner in one sitting<br>- Fewer exams per patient on average due to a findings-based screening interval	- Ability for consultation using the raw image data<br>- Scalability<br>- Screener constancy (no changing of graders)<br>- Mandated screening intervals limits the possibility of a name mix-up with multiplets and different screening follow up intervals
Limitations	
- Screening is difficult (requires a skilled Pediatric Ophthalmologist or Retina Specialist) and is time-consuming<br>- Subjective grading - it represents an interpretation of what the examiner noted and then subsequently documented on a cartoon schematic.<br>- No ability to directly compare examinations longitudinally in one sitting or between graders. The only thing that can be compared longitudinally is the one examiner's subjective interpretation.<br>- If there is inadequate dilation, an unstable infant, improper equipment, the examination will be suboptimal.<br>- Findings-based intervals may lead to improper disease assessment in patients with similar names or multiplets with different levels of disease.	- Potential for ungradable photos which means the infant must undergo BIO screening<br>- Expensive up-front camera cost ($30,000-150,000)<br>- Camera lens must make contact with the eye to obtain focus<br>- Requires a skilled photographer that follows a protocol imaging set<br>- 130° five-field views from a fundus camera only image about 95% of the retina<br>- Lack of three-dimensionality<br>- More exams per patient on average due to a weekly mandated screening interval<br>- If there is inadequate dilation, an unstable infant, improper equipment, the examination will be suboptimal.
