# Supplementary material for: Cost-Effectiveness of Telemedicine-Based Screening Programs for Retinopathy of Prematurity in Low- and Middle-Income Countries
Source: Ophthalmol Sci. 2026 Sep 4;6(11):101395. doi: 10.1016/j.xops.2026.101395 (PMC13654025; doi:10.1016/j.xops.2026.101395)
Supplement: Supplementary file 2 [file mmc2.pdf]

Supplemental Table 2.

Strategy	Costs ($)	QALYs		ICERs ($/QALY)	
**Utility Case**	**Both[18,19]**	**Best[18]**	**Worst[19]**	**Best[18]**	**Worst[19]**
Standard of Care	0	399,712	395,993	--	--
Telemedicine screening (Non-Physician)	102,835	401,778	401,526	50	19
Ophthalmoscopy	426,414	401,778	401,526	Dominated	Dominated

1.5% Discount: *Costs, Quality Associated Life Years (QALYs), and Incremental Cost effectiveness ratios (ICERs) by strategy grouped by best- and worst-case utility values for blindness. Dollar amounts in 2025 USD for 10,000 Patients*

Strategy	Costs ($)	QALYs		ICERs ($/QALY)	
**Utility Case**	**Both[18,19]**	**Best[18]**	**Worst[19]**	**Best[18]**	**Worst[19]**
Standard of Care	0	648,358	642,325	--	--
Telemedicine screening (Non-Physician)	102,835	651,709	651,302	31	11
Ophthalmoscopy	426,414	651,709	651,302	Dominated	Dominated

0% Discount *Costs, Quality Associated Life Years (QALYs), and Incremental Cost effectiveness ratios (ICERs) by strategy grouped by best- and worst-case utility values for blindness. Dollar amounts in 2025 USD for 10,000 Patients*
