# Supplementary material for: Cost-Effectiveness of Telemedicine-Based Screening Programs for Retinopathy of Prematurity in Low- and Middle-Income Countries
Source: Ophthalmol Sci. 2026 Sep 4;6(11):101395. doi: 10.1016/j.xops.2026.101395 (PMC13654025; doi:10.1016/j.xops.2026.101395)

Supplemental Figure 1. *Cost-effectiveness plane including standard of care, Telemedicine with n% physician grading (0-100% in 10% increments), and ophthalmoscopy with worst-case utility values. QALYs: Quality Adjusted Life Years. Costs are expressed in US Dollars.*

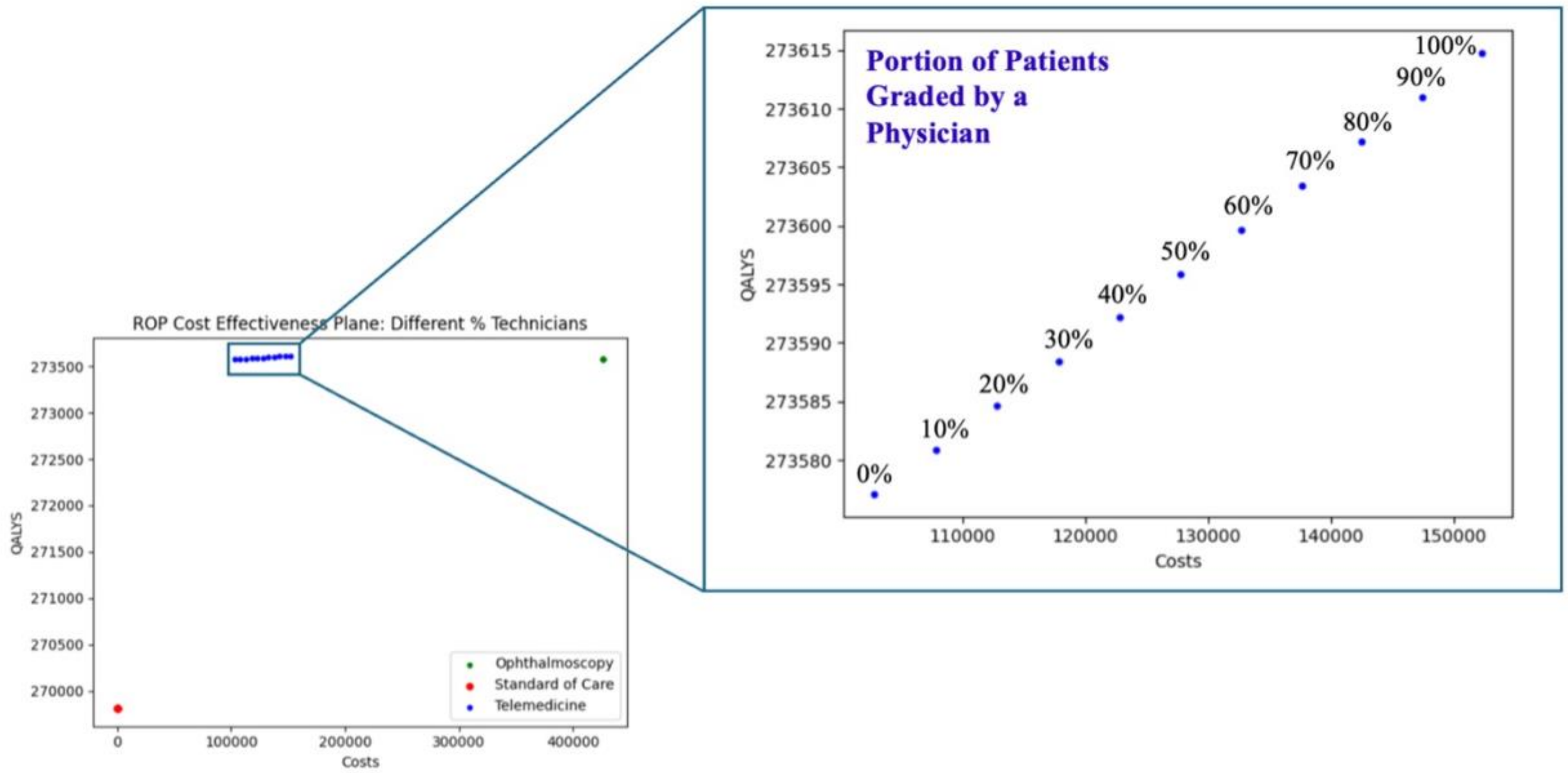

Supplement: Supplementary file 3 [file mmc3.pdf]
