# Supplementary material for: Cost-Effectiveness of Telemedicine-Based Screening Programs for Retinopathy of Prematurity in Low- and Middle-Income Countries
Source: Ophthalmol Sci. 2026 Sep 4;6(11):101395. doi: 10.1016/j.xops.2026.101395 (PMC13654025; doi:10.1016/j.xops.2026.101395)

Supplemental Figure 2. *Stacked one-way sensitivity analyses showing how the most cost-effective strategy varies as one parameter at a time is changed.*

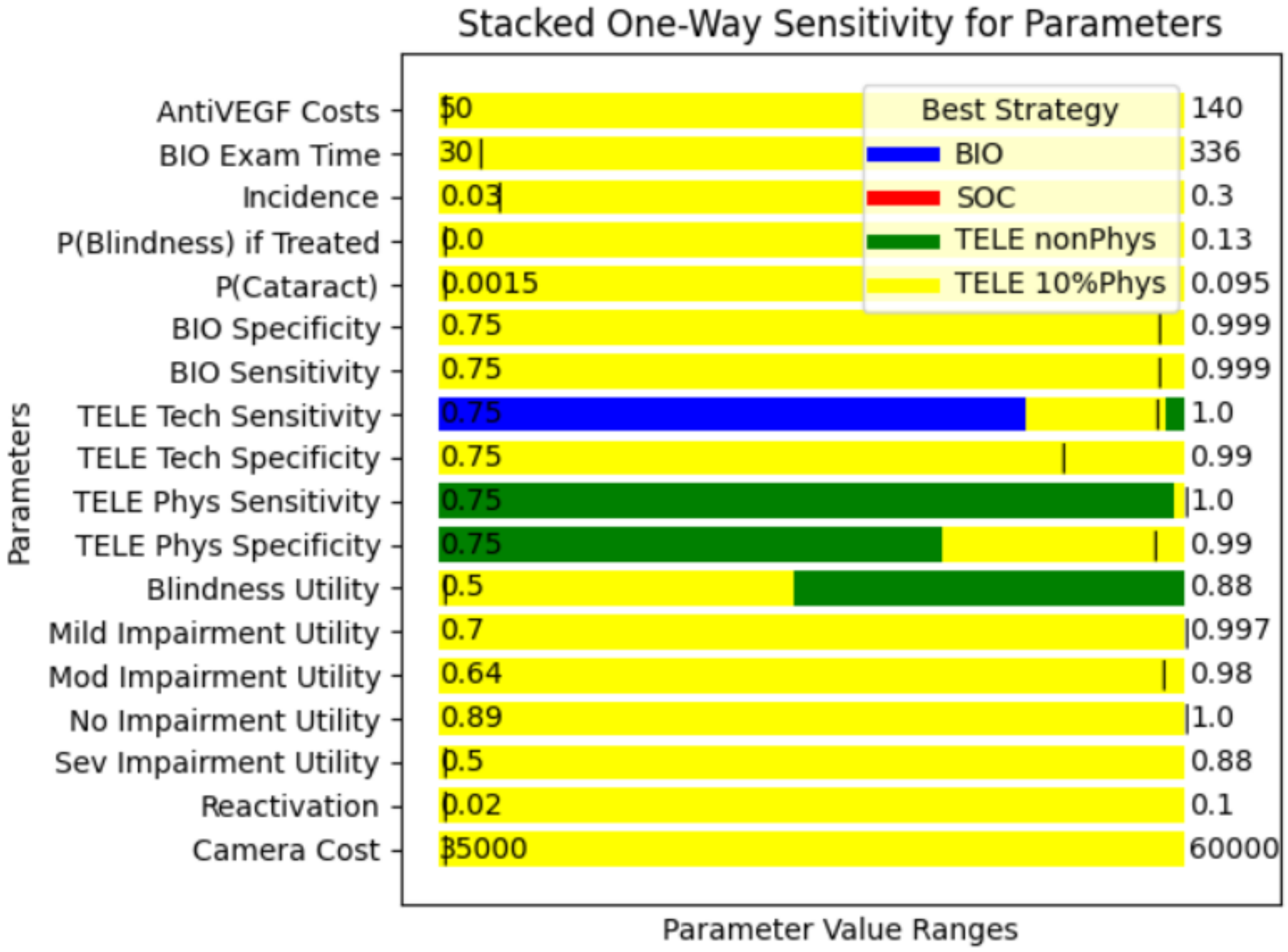

Supplement: Supplementary file 4 [file mmc4.pdf]
