# Supplementary material for: Cost-Effectiveness of Telemedicine-Based Screening Programs for Retinopathy of Prematurity in Low- and Middle-Income Countries
Source: Ophthalmol Sci. 2026 Sep 4;6(11):101395. doi: 10.1016/j.xops.2026.101395 (PMC13654025; doi:10.1016/j.xops.2026.101395)

Supplemental Figure 3. *One-way sensitivity analysis showing the variation in net monetary benefit (NMB) in relation to the base case (dotted line) with A) variation in technician grader sensitivity and B) technician specificity. Solid lines represent the most cost-effective strategy at each NMB increment.*

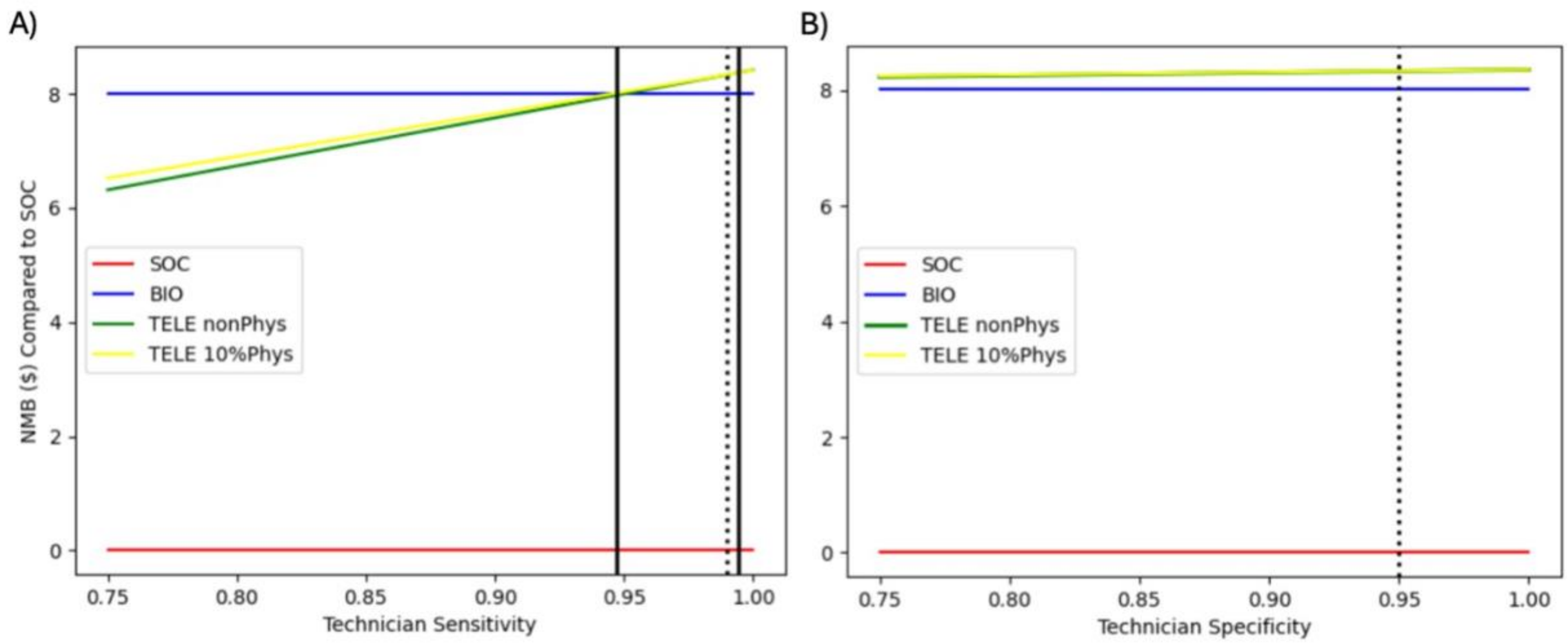

Supplement: Supplementary file 5 [file mmc5.pdf]
