# Supplementary material for: Cost-Effectiveness of Telemedicine-Based Screening Programs for Retinopathy of Prematurity in Low- and Middle-Income Countries
Source: Ophthalmol Sci. 2026 Sep 4;6(11):101395. doi: 10.1016/j.xops.2026.101395 (PMC13654025; doi:10.1016/j.xops.2026.101395)

Supplemental Figure 4. *Two-way sensitivity analysis showing the variation of sensitivity and specificity of a technician grader. The 'plus' indicates the base case. The best strategy was assessed by a willingness-to-pay threshold of 1X GDP per capita of Ghana.*

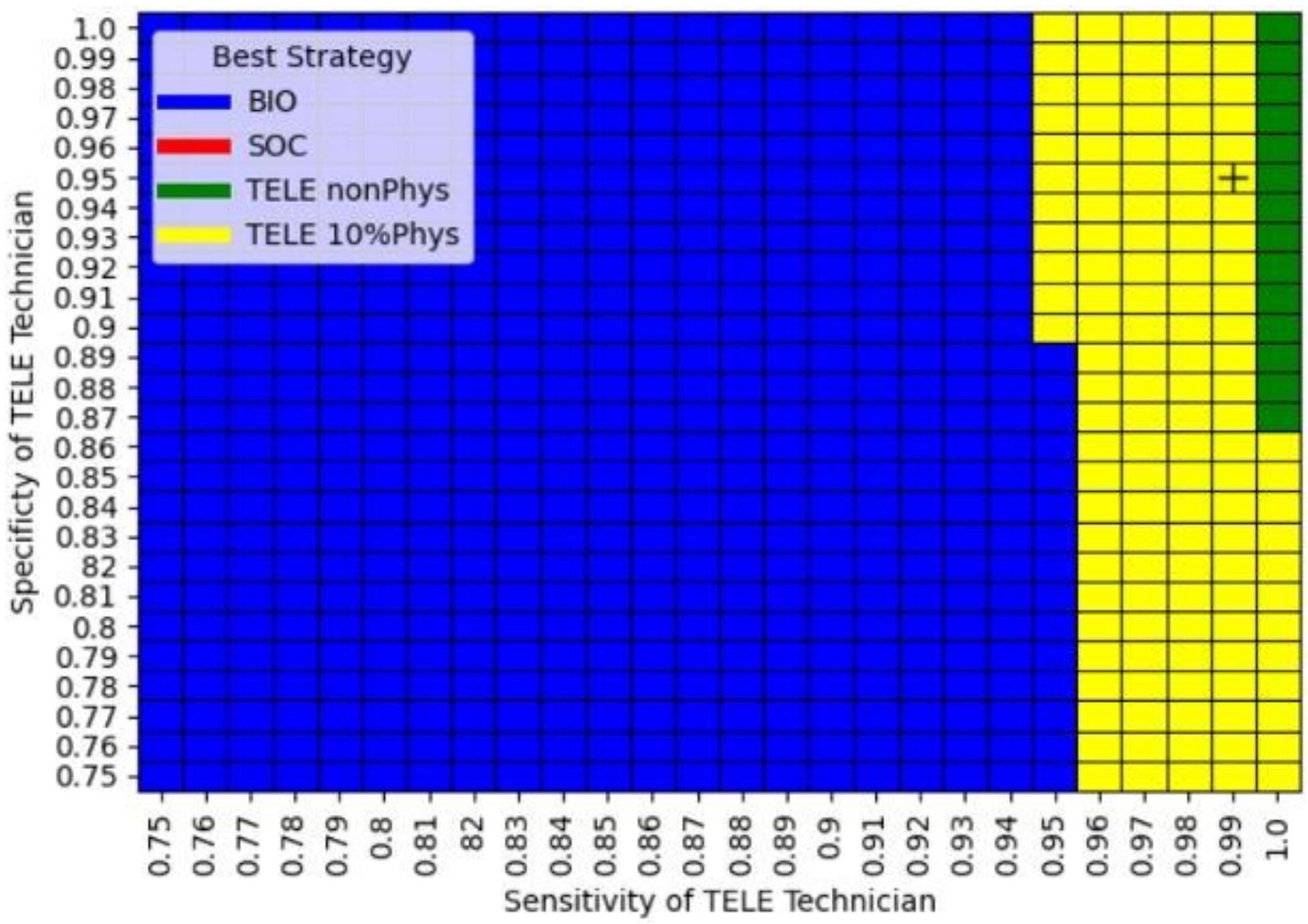

Supplement: Supplementary file 6 [file mmc6.pdf]
